# A novel proprioceptive rehabilitation program: A pilot randomized controlled trail as an approach to address proprioceptive deficits in patients with diabetic polyneuropathy

**DOI:** 10.1371/journal.pone.0305055

**Published:** 2024-07-05

**Authors:** Kavinda T. Malwanage, Esther Liyanage, Vajira Weerasinghe, Charles Antonypillai, Indumathie Nanayakkara

**Affiliations:** 1 Department of Physiotherapy, Faculty of Allied Health Sciences, University of Peradeniya, Peradeniya, Sri Lanka; 2 Department of Physiology, Faculty of Medicine, University of Peradeniya, Peradeniya, Sri Lanka; 3 Diabetes and Endocrinology Unit, National Hospital, Kandy, Sri Lanka; PearResearch / Government Doon Medical College, INDIA

## Abstract

**Background:**

Diabetic polyneuropathy (DPN) is a notable microvascular complication of DM, affecting 16%-66% globally. DPN often leads to proprioceptive deficits in the lower limbs (LL), leading to impaired functional performance. However, evidence supporting proprioceptive rehabilitation programs (PRP) for DPN remains scarce.

**Aims:**

This pilot study aims to evaluate the effectiveness of a novel 12-week PRP on LL static and dynamic proprioception and shed light on the potential benefits of PRP for DPN population.

**Methods:**

Randomized Controlled Trail was conducted among 30 DPN patients (age 53.25±7.72 years, BMI 24.01±1.41 and DM duration 9.48±6.45 years), randomly allocated to *intervention* (n = 15) or *control* (n = 15) groups. The intervention group received PRP 3 times/week for 12 weeks. The control group received no exercise. Both groups received regular diabetic care. Static and dynamic proprioception of both LL were assessed at baseline, 6 weeks and 12 weeks. Position-reposition test was used to assess ankle joint position sense by obtaining difference between target and reproduced angles. Error in detecting knee angle and speed were obtained by performing Lower Limb Matching and Sense of Movement tests respectively to assess dynamic proprioception.

**Results:**

Two-way ANOVA and paired comparisons revealed, no significant improvement in proprioceptive deficits at 6 weeks (*p*>0.05), but significant improvement was achieved at 12-weeks (*p*<0.05) in the intervention group. Mean errors in Pposition re-position(R:*p*<0.001, L;p<0.001) and Lower limb matching (R:p<0.001, L;p<0.001) tests reduced by 5° and 10° respectively, indicating a70% improvement in the intervention group. Error of detecting speed reduced only on right side by 0.041ms^-1^ accounting for a 42% improvement. No improvements were observed in the control group.

**Conclusions:**

Novel 12-week PRP may yield a significant reduction in LL proprioceptive deficits among DPN patients. Future RCTs with larger samples should compare the effectiveness of this PRP compared with conventional rehabilitation programs.

## Introduction

Diabetes mellitus (DM) has become the most rapidly escalating global health emergency of the 21^st^ century reporting a higher incidence of mortality and morbidity rates worldwide [1]. Diabetic polyneuropathy (DPN), characterized by peripheral nerve dysfunction is one of the notable microvascular complications of DM which affect approximately 16%-66% of the global population [2, 3]. In DPN, damage occurs to the sensory and motor nerves in response to long-term elevation of blood typically starting distally in the feet and progressing proximally to hands. Since DPN is a diagnosis of exclusion, screening for signs and symptoms of DPN enables early intervention to improve symptoms and reduce sequelae [2].

Neuropathic pain, dysesthesias, and hyperalgesia are frequently experienced symptoms that arise from the involvement of small fibers, while numbness, tingling sensations, and impaired vibration and proprioception stem from the involvement of large fibers in DPN. Proprioceptive impairments have a direct impact on daily functioning, owing to the fact that the roots of every functional activity are ground beneath ‘proprioception’.

Proprioception is the ability of the individual to perceive joint position sensation i.e., appreciation and interpretation of information related to the joint position and orientation in space [4]. It also provides multifaceted details on the sensation of force generation, sense of effort and awareness of the body in space [5] to execute effective and efficient daily activities. Hence, proprioception enables precise, effective and efficient functional movements as it involves unconscious processing of somatosensory information.

Accurate integration and processing of movement patterns during functional activities rely on appropriate proprioception function. Conversely, proprioceptive deficits result in erroneous feedback mechanisms on motor control leading to ineffective and inefficient movements.

Multiple lines of evidence suggest that DPN patients exhibit lower limb proprioceptive deficits when compared to the healthy control resulting in significant changes in motor control, coordination, and overall body awareness [6, 7]. Moreover, it was found that DPN patients have a thicker Achilles tendon [8], stiffer plantar tissue [8] and Charcot neuroarthropathy [9] which may collectively alter the biomechanical properties of the soft tissue of ankle-foot complex [10] and foot kinetics and kinematic parameters [11]. Therefore proprioceptive deficits combined with motor impairment contribute to gait abnormalities, impaired balance and coordination, alteration of postural stability, and increased risk for falls [12, 13] contributing to poorer physical performance, particularly among adults with DPN.

Skilful movement may be impossible or erroneous without proper proprioceptive function in DPN leading to functional disability. Some performance-based tasks i.e. walking, standing, bending, mobility etc. [14] stair-climbing and sit-to-stand [15] were found to be affected by the neuropathy status in adults with DPN leading to poorer physical functioning. Consequently, this limitation in activity leads to participation restriction depending on the severity of proprioception deficit. It is evident that proprioception deficits combined with other complications of DPN lead to a considerable decline in quality of life in DPN patients [16, 17].

There are various treatment approaches viz pharmacotherapy [2, 18], dietary [19], lifestyle modifications [20] and physical therapy to address issues associated with DPN. Various forms of physical therapy interventions have demonstrated their ability to yield sustainable and efficient clinical outcomes while mitigating the progression of DPN-related complications [21, 22]. These encompass aerobic exercises, resistance training, balance training, combined training i.e. aerobic and resistance, walking programs, Tai Chi and electrotherapy [23–26].

Given the importance of proprioception for motor control, it has been argued that therapies aiming to restore motor function after injury should focus on training the proprioceptive sense. Proprioceptive training is found to be effective in improving somatosensory and sensorimotor functions by inducing cortical reorganization in people with neurological diseases [4]. In the context of DPN, sensorimotor training is found to be one of the effective non-pharmacological interventions to improve DPN-related dysfunctions with significant improvement in static balance, gait, nerve conduction velocity and HbA1c [27].

Eight weeks of proprioceptive training program along with conventional physiotherapy were proven to be effective in improving functional balance in patients with DPN [28]. A study conducted by Santos et al. in 2008 found that 12 weeks of proprioceptive training were effective in increasing plantar tactile sensitivity and reducing anterior-posterior oscillations of the center of the pressure in the female patients with DM [29]. Further, it was found that 8 weeks of ankle proprioceptive training was effective in improving gait and reduce risk of falling in patients with DPN [30]. Ahmad et al. (2020) found that 8-week of sensorimotor training combined with gait training improves trunk proprioception in DPN patients.

Accordingly, it is evident that only a few studies have employed proprioceptive training either independently or in combination with other therapeutic techniques to evaluate its effectiveness in improving trunk proprioception, gait and balance impairment in patients with DPN although the effect on postural stability and QoL has not been studied comprehensively.

The founder of sensorimotor rehabilitation, Janda et al. (1996) emphasized the importance of increasing proprioceptive input by improving afferent information into CNS to facilitate automatic coordinated movements [31]. Sensorimotor training involves progressively stimulating sensory inputs through specifically designed exercises with increasing levels of challenge of the task using a functional, rather than a structural approach [31, 32].

Therefore it is noteworthy that proprioceptive sensory inputs are essential for accurate muscular balance for a correct motor programming [33]. Hence, the goal of sensorimotor training is to increase proprioceptive input to stimulate subcortical pathways and facilitate automatic coordinated movements [33].

Janda introduced two basic stages of motor re-learning in rehabilitation, emphasizing voluntary control of movement in the first stage, requiring cortical regulation through higher concentration. Constant feedback is required during this stage. When new coordinated movement patters are learned, it is thought to be programmed in the subcortical region, becoming more ‘automatic’, requiring less conscious effort to perform same exercise thereafter. It results in ‘Feed-forward mechanisms’ to activate unconsciously preparing the body for proper movements. This automatic level of movement processing is considered crucial for effective daily functions, achievable through sensorimotor training.

Due to the abundance of proprioceptors in the foot, foot proprioception is considered as a key determinant of many functional movements [34]. Therefore it was found that input should be provided to the sensorimotor system ‘from the ground-up’ mechanism [32]. Proper positioning and movements of foot hence be ensured during each and every exercises of sensorimotor training. In addition, Freeman and Wyke (1967) emphasized that barefoot is the best to perform proprioceptive exercises which ensures the maximum amount of afferent information entering the sensorimotor system [35]. This fact emphasize that sole of the foot should be stimulated with sensory inputs, and the movement of foot and ankle joints should also be encouraged.

To induce proprioception, small repetitive exercises should be performed with gradual increase in the difficulty to enhance the recruitment of proprioception [36]. Repetitive activities using familiar objects and tasks are believed to improve adaptability of sensorimotor system [37]. To further improve the adaptability, novel objects and advanced tasks should also be introduced later in the rehabilitation program.

Sensorimotor rehabilitation programs designed based on these concepts have been proven to yield positive outcomes on proprioception, strength and postural stability in patients with DPN [38, 39]. Therefore, many studies suggested implementing proprioceptive training, in addition to strength training and balance training among patients with DPN to regain the functional capacity [40].

Nevertheless, there remains a lack of evidence supporting properly devised lower limb proprioceptive rehabilitation programs which specifically improves the key elements of proprioception viz static and dynamic proprioception in patients with DPN. Among healthcare professionals, physiotherapists have the potential to make a valuable contribution by designing simple yet comprehensive rehabilitation programs, to address proprioceptive deficits, thereby promoting recovery and restoring optimal physical functioning.

Despite the strong relationship between DPN and proprioceptive deficits, evidence supporting lower limb proprioceptive rehabilitation in DPN is sparse. The novel PRP is suggested to fill the gap by providing the scientific evidence to physiotherapists.Therefore, this study aimed to evaluate the effectiveness of a novel 12-week proprioceptive rehabilitation (PRP) on lower limb static and dynamic proprioception among patients with DPN.

## Materials and method

### Study design and setting

This double blinded randomized controlled trial was conducted as a pilot study of a large study among the patients with DPN who visited, Diabetic and Endocrinology Clinic, Kandy National Hospital during October 2023 to December 2023.

Ethical clearance was obtained from the Ethics review committee, Faculty of Medicine, University of Peradeniya (2023/EC/20). The study was also registered under Sri Lanka Clinical Trial Registry with the registration number of SLCTR/2023/017 to conduct as a clinical trial in Sri Lanka.

A total of 30 patients were required to validate 12-week PRP, employing the ANOVA test under the F-test. The limited sample size is justified for this pilot RCT to ensure the assessment of the feasibility of the study protocol, including recruitment, retention and adherence to the intervention.

The investigator visited Diabetic and Endocrinology clinic and meet the patients with diabetic polyneuropathy individually one at a time. Then each patient was assessed against predefined inclusion and exclusion criteria to determine their eligibility for participation in the study. Upon confirmation of eligibility and obtaining the written informed consent, the selected patient was randomly assigned to either intervention or control groups by applying simple randomization method. Concealed envelops were used to effectively conceal the randomization ensuring allocation concealment, preventing selection bias and also ensuring that participants and investigators remain unaware of the treatment assignment until after allocation. For this randomization, the random allocation sequence was generated and it remained concealed in sequentially numbered, opaque envelopes which were assignment sealed. Participant drew a number in sequence and patient’s reference number was noted on the envelope before tearing it. Lastly, the envelop was opened to disclose the treatment allocation. Participant and the assessor were blind from group allocation. This was carried out to until the required sample size achieved i.e 30 participants. In this procedure, each participant has equal chance to be selected for one of the two groups, no any favor was offered. The procedure was carried out by the principle investigator.,

Recruited participants were instructed to visit the Department of Physiology, Faculty of Medicine, University of Peradeniya at the baseline, 6 weeks after the commencement and at the end of 12-weeks to undergo the assessment and treatment. Static and dynamic proprioception of both lower limbs were assessed in each participant.

### Participants

**Fig 1:**
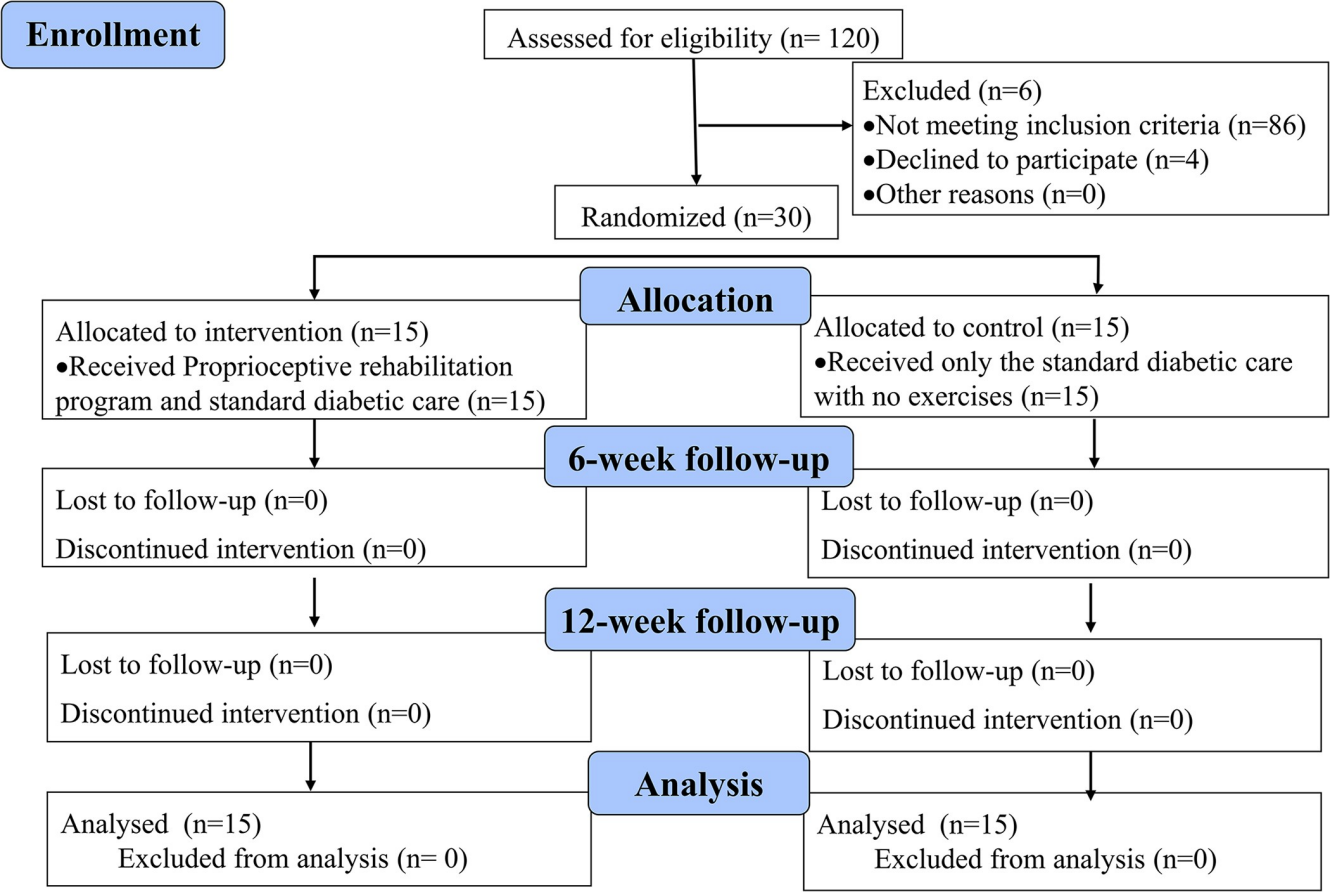
CONSORT flow diagram of the progress of RCT.

One hundred and twenty patients with type-2 DM were screened and 30 patients with DPN were included in the study. The patients were included if (1) they had been on regular treatment for type-2 diabetes mellitus for one or more than one year; (2) within the age group of 35–60 years; (3) having DPN and (4) provided informed written consent for participating in the study. Patients with acute cardiovascular diseases, autonomic dysfunctions, recent surgery, severe pain and paresthesia, other types of neuropathy, diabetic foot ulcers, impaired vision, acute lower limb injury, significant psychiatric disorders, amputation, obese patients, pregnant women together with the patients who did not give consent were excluded. The participant selection and progress of the RCT are presented in Fig 1.

### Identification of DPN

Validated and reliability-tested Sinhala and Tamil versions of the Michigan Neuropathy Screening Instrument (MNSI) were used to identify the presence of DPN in the patients. The MNSI consisted of two sections viz a self-administered questionnaire completed by the patient (section A) and a physical assessment conducted by a medical professional (section B) [41, 42].

Section A assessed the DPN clinical symptoms via 15 yes/no type questions. All questions except 7 and 13 were scored as 1 point if answered ‘yes’; questions 7 and 13 were scored as 1 point if answered ‘no’.Question 4 was related to impaired circulation and question 10 was related to general asthenia and neither was included in the original MNSI scoring.

Section B involved a physical assessment of the feet for dry skin, calluses, infections, fissures, and ulcers, followed by an evaluation of ankle reflexes and vibration perception. Each foot with a deformity was counted as 1 point and each foot with an ulcer was also counted as 1 point. Ankle reflex was scored as 0 if present, 0.5 points only when the patient performed the Jendrassik maneuver, and 1 point if absent despite the Jendrassik manoeuvre. Vibration perception was assessed using a 128-Hz tuning fork at the distal interphalangeal joint of the participants’ great toe. The score was determined based on how much longer the examiner could feel the vibration than the patient. If the examiner felt the vibration for 10 or more seconds longer than the patient, vibration sense was considered decreased and scored as 0.5 points. If the examiner felt the vibration for less than 10 s longer, vibration sense was considered normal (0 points). If the patient could not sense the vibration at all, they received 1 point.

Patients who scored greater than 4 points in the self-administered questionnaire (Part A) and greater than 2 points in the physical examination part (Part B) of MNSI, were diagnosed to have DPN [41, 43, 44].

### Study protocol and intervention

On the first day, demographic data i.e. age, gender, height, weight, co-morbidities and duration of DM were recorded and lower limb static and dynamic proprioceptive impairment were evaluated in all the participants. Exercises of the proprioceptive rehabilitation program were taught to the intervention group by the principal investigator, which was then carried out by the participants at the department and corrected as required. Furthermore, participants in the intervention group were provided a printed exercise protocol along with bi-weekly progression, containing step-by-step instructions accompanied by illustrations, to enable them to continue at home. Participants’ compliance with the exercise progression was assessed through phone calls. Participants in the control group were not administered any exercises. Both groups received regular diabetic care from the hospital. The novel, lower limb proprioceptive rehabilitation program involved exercise sessions lasting for 40–45 minutes, conducted 3 times per week over 12 weeks. Each session included three sub-sessions: a warm-up session, proprioception training exercises and a cool-down session. This novel proprioceptive rehabilitation program involved various exercise categories such as spatial orientation, static balance, dynamic balance, stability challenge, reaction time, fine motor skills and coordination exercises in the lower limb which were designed to adhere to the basic principles of neuromuscular rehabilitation i.e., participant’s concentration, rationalization, feedback, active exercises, and repetitions of movements [45]. These elements were found to act as adaptive codes to improve neuromuscular adaptation by recruiting proprioceptors.

The rehabilitation protocol was devised to increase the difficulty of the exercises every 2 weeks throughout the designated 12-week period. As the training progressed, each exercise was modified to make them more demanding by removing visual feedback (eyes closed) and changing the support surface, body position, direction, speed, and distance. The detailed protocol is shown in S1 File.

### Tests and measurements

Outcome measures of interest involved static and dynamic proprioception of both lower limbs which was assessed at the baseline, six weeks after the commencement and at the end of 12-week rehabilitation program. The ‘Position re-position test’ was employed to assess static position sense by calculating the absolute mean error in angle (degrees). Meanwhile, the evaluation of dynamic movement sense involved the ‘Lower limb matching test’, which was assessed using the mean angle difference in degrees and the ‘Sense of movement test’ which was assessed by mean speed difference in meters per second (m/s). Accordingly, all the outcome variables utilized ratio-level measurement scales. Prior to each test, participants were familiarized with the tests by giving clear and accurate instructions. Tests were performed in the absence of visual and auditory cues.

#### Assessment of static proprioception

‘Position-reposition test’ was performed to evaluate the static position sense in each ankle joint which assessed the ability to accurately perceive joint position [45]. During the test, participant was seated with eyes closed on a high couch with the knees flexed at 90° and the lower leg positioned vertically. Investigator set the participant’s ankle joint to a pre-determined angle and held that position for 2–4 seconds to allow the participant to feel and remember the target position followed by passively moving the ankle into starting position. Then, participant was instructed to reproduce the angle at the ipsilateral ankle joint. Each test was followed by a 15-second rest period. Absolute mean error which is also called as position sense error (i.e. the difference between the pre-determined/target and reproduced angles measured in nearest 0.1° degrees) of three trials was used as the measure of static position sense.

#### Assessment of dynamic proprioception

‘Lower limb matching test’ and ‘Sense of movement test’ were carried out to determine the dynamic proprioception [45]. During the tests, the participant was seated on a high couch with eyes closed, the knees flexed at 90° and the lower leg positioned vertically. To test the ability to perceive the movement sense, the investigator moved one limb and instructed the participant to follow the same movement with the contralateral leg at the same angle and same speed. The measurements of the difference of the knee angles were obtained in degrees to the nearest 0.01°. Mean difference between target and reproduced knee angles and mean speed differences between both lower limbs in three trials were calculated to obtain the outcome measure for dynamic proprioception.

The two testing procedures were recorded using a high-speed video camera in the sagittal plane with the test limb facing the camera. Camera was mounted on a tripod. The videography was taken at the rate of 30 frames per second with a screen resolution of 720×1080 pixels in an MP4 file. To facilitate the identification of anatomical landmarks on the video, the human body markers with a 1.5 cm diameter were placed on the greater trochanter, lateral epicondyle, and lateral malleolus of the participants’ test limbs.

Kinovea 0.9.5 motion analysis software (Joan Charmant & Contributors, 2006) was used to analyze and record the target angle and reproduced angles formed by lines connecting the centres of the body markers in degrees to the nearest 0.1°. Similarly, the speed of each lower limb was evaluated using the linear kinematic analysis option in Kinovea software. Mean difference of speed of three different random positions of each lower leg was recorded to obtain ‘mean speed difference’ as the outcome measure of ‘sense of movement test’. The speed was obtained from pixels per second (px/s) which was then transformed to meters per second (m/s) to report the speed in SI unit. All the video graphics measurements were evaluated and obtained by another assessor who was a qualified physiotherapist and was blinded to the group allocation.

Higher differences/ error values corresponded to the lower proprioception function.

### Statistical analysis

Data from 30 participants from intervention group (n = 15; 6 males, 9 females) and control group (n = 15; 4 males, 11 females) were included in the analysis. Absolute mean difference for position repositioning test, mean angle difference for lower limb matching test and mean speed difference for sense of movement tests obtained at three-time points i.e., baseline, 6 weeks and 12 weeks were compared between the intervention and control groups to investigate the effectiveness of the novel proprioceptive rehabilitation program on proprioception compared to no intervention.

Baseline data was compared between the two groups to investigate the normality and homogeneity of variance across all the variables. Two-way, group (intervention vs. control) × time (pre, mid, post) mixed analysis of variance (ANOVA) was conducted on three outcome measures to evaluate the overall effect of proprioceptive rehabilitation on proprioceptive deficits compared to control. Further, post-hoc analysis were performed to investigate whether the treatment duration i.e., 6 weeks and 12 weeks had an influence on the improvement in lower limb proprioception. Additionally, to test whether the outcome measures were statistically significantly different after adjusting the effect of two covariates i.e. gender and side, covariate analysis was also carried out. The level of significance was ascertained at a cut-off *p*-value of less than 0.05. All statistical analyses were performed using IBM SPSS 22.0 software (Armonk, NY: IBM Corp).

## Results

### Characteristics of the participants

This study included 30 patients with DPN with a mean age of 53.23 (SD = 7.79) years, mean height of 159.08 (SD = 8.95) cm, mean weight of 61.11 (SD = 8.15) kg, mean Body Mass Index (BMI) of 22.05 (SD = 1.03) kg/m^2^ and mean DM duration of 9.43 (SD = 6.12) years. Groups were similar at baseline, with no significant differences in demographic or clinical characteristics. Dependent variables exhibited a normal distribution (*p*>0.05) and homogeneity of variance (*p*>0.05) across both groups as verified by Shapiro-Wilk test and Levene’s test respectively. Table 1 presents the characteristics of the intervention and control groups.

**Table 1:** Demographic characteristics of the sample at the baseline.

Measure	Intervention group (n = 15)	Control group (n = 15)	Mean difference [95% CI]
Age (years) (mean ± SD)	54.73±7.99	51.73±7.54	3.00 [-2.81, 8.81]
Height (m) (mean ± SD)	1.61±0.08	1.58±0.09	0.03 [-0.04, 0.10]
Weight (kg) (mean ± SD)	62.02±8.78	60.20±7.65	1.82 [-4.33, 7.99]
BMI (kg/m^2^) (mean ± SD)	23.95±1.36	24.16±1.44	-0.21 [-1.25,0.85]
Duration of DM (years) (mean ± SD)	9.27±6.25	9.60±6.79	-0.33 [-5.21, 4.55]
*Diagnosis of MNSI*			
MNSI-Part A	7.07±2.09	7.47±1.77	-0.40[-1.85,1.05]
MNSI-Part B	4.73±1.45	6.07±1.20	-1.33 [-2.30,-0.37]

*Note*. BMI = Body Mass Index, DM = Diabetes Mellitus, MNSI = Michigan Neuropathy Screening Instrument

### Attrition, adherence, and adverse events

In this study, participant attrition, adherence to the study protocol and monitoring of adverse events were observed. The attrition rate of the study was 100% with all 30 enrolled participants completing the rehabilitation program by the end of the study. Adherence to the intervention was monitored through weekly phone calls and participants’ self-reports, wherein they were instructed to mark the relevant grid in the provided table upon session completion. As the outcome measure of study involved obtaining the measurements in sitting positions, and the study intervention involved simple exercises, no adverse event was reported during the study period. However, participants’ complaints related to other symptoms were documented and conveyed to the relevant medical practitioners.

### Effect of the PRP on lower limb static and dynamic proprioception

The outcome of two-way time (pre, post) × group (intervention, control) ANOVA and paired comparisons of static and dynamic proprioception are summarized in Table 2. The results revealed significant group, time (pre, mid, post) and time* group interaction effect following 12-week PRP in the intervention group. Accordingly, absolute mean error of position re-position test (right:p<0.001, left;p<0.001) and mean angle difference of lower limb matching test (right:p<0.001, left;p<0.001) were significantly reduced in the intervention group following the 12-week rehabilitation program. Regarding sense of movement test, lower limb speed error was significantly reduced only in the right side (p< 0.01) but not for the left side (p = 0.54). Only the group effect (p< 0.01) was significant for the sense of movement in left lower limb which is suggestive of a positive trend for the improvement. Neither the mid-assessment nor the final assessment showed any signs of improvement in the control group (p>0.05).

**Table 2:** ANOVA and paired comparisons of lower limb proprioception.

Outcome measure	Two-way ANOVA significance (p values)			Within-group pre-post comparisons					
				Intervention group			Control group		
	Group	Time	Time× Group	Pre-test mean±SD	Post-test mean±SD	Difference [95%CI], *p* value	Pre-test mean±SD	Post-test mean±SD	Difference [95%CI], *p* value
Position-reposition test: *Absolute mean error (°)*									
Right	<0.001*	<0.001*	<0.001*	8.07±1.83	2.42±1.26	5.65 [4.61, 6.70], <0.001*	7.23±1.37	7.91±1.50	-0.69 [-1.97, 0.60], 0.27
Left	<0.001*	<0.001*	<0.001*	6.87±1.50	2.18±0.51	4.69 [3.77, 5.61], <0.001*	7.62±1.97	7.17±2.82	0.45 [-1.06, 1.96], 0.53
Lower limb matching test: *Mean angle difference (°)*									
Right	<0.001*	<0.001*	<0.001*	13.48±0.87	4.36±0.41	9.12[7.11,11.13], <0.001*	15.26±3.75	16.96±3.81	-1.70[-4.56, 1.16], 0.22
Left	0.03*	<0.001*	<0.001*	14.04±3.97	3.93±2.03	10.11[7.79,12.44], <0.001*	14.25±4.20	19.90±5.35	-5.65[-9.62,-1.68], 0.07
Sense of movement test: *Mean speed difference (ms*^*-1*^*)*									
Right	0.75	0.02*	0.002*	0.098±0.034	0.057±0.024	0.041[0.019,0.064], 0.002*	0.085±0.026	0.101±0.033	-0.016[-0.038,0.005], 0.12
Left	0.004*	0.82	0.20	0.076±0.032	0.067±0.046	0.001[-0.021,0.039], 0.54	0.082±0.034	0.094±0.025	-0.013[-0.031,0.006], 0.16

*p < 0.05, compared with control group

Post-hoc analysis revealed, non-significant improvement of proprioceptive deficits at the mid-assessment (p>0.05), but significant improvement following 12-week proprioceptive rehabilitation program in the intervention group (p<0.05). Further, post-hoc analysis showed only significant pre-post differences (T1-T3) in the intervention group on both sides for the position re-position test (right:p<0.001, left;p<0.001) and lower limb matching test (right:p<0.001, left;p = 0.01) indicating reduction of proprioceptive deficits only in the intervention group. Pre-post error reduction was not significant for the sense of movement test neither in intervention group nor control group.

Pertaining to the within-group pre-post comparisons, there was a significant reduction in absolute mean error during static positon sense test in both the right (*t* (14) = 8.88, *p* < 0.001) and left (*t* (14) = 8.53, *p* < 0.001) sides, corresponding to a decrease of 5.65° and 4.94° angle error respectively in identifying lower limb static position sense among the participants in the intervention group. Conversely, no significant improvement was observed in the control group for either the right (*t* (14) = -0.17, *p* = 0.27) or left (*t* (14) = 1.06, *p* = 0.53) side static proprioception following no intervention.

In the pre-post test comparison of mean angle difference during Lower limb matching test, the intervention group significantly improved the ability to detect lower limb movement on both the right (*t* (14) = 6.99, *p* < 0.001) and left (*t* (14) = 6.93, *p* < 0.001) sides, resulting in respective error reduction of 9.12° and 10.11° following the 12-week PRP. Nevertheless, control group exhibited a further increase in error in detecting lower limb movement on the right (*t* (14) = -1.43, *p* = 0.22) and left (*t* (14) = -1.24, *p* = 0.07) sides.

Regarding the sense of movement test, there was a significant reduction in mean speed difference on the right side (*t* (14) = 3.29, *p* = 0.002) by 0.041 m/s following the 12-week PRP. However, no such significant improvement was observed for the left side (*t* (14) = 4.23, *p* = 0.54). The control group showed further deterioration of detecting lower limb speed by 0.016 m/s (*t* (14) = -1.43, *p* = 0.12) and 0.013 m/s (*t* (14) = -1.24, *p* = 0.16) on the right and left sides respectively. The overall results suggest a reduction of mean error nearly by 5° which attributes to 70% improvement from the pre-intervention static proprioceptive deficits of lower limbs. Similarly, mean angle difference reduced significantly in the intervention group by approximately 10°, amounting to an improvement by 70% from pre-intervention dynamic proprioceptive deficit. Additionally, right side sense of movement test showed a 0.041ms^-1^ reduction in mean speed error, accounting to 42% improvement from pre-intervention value for dynamic sense of movement. However, left side mean speed error did not reduce significantly in the intervention group.

**Fig 2:**
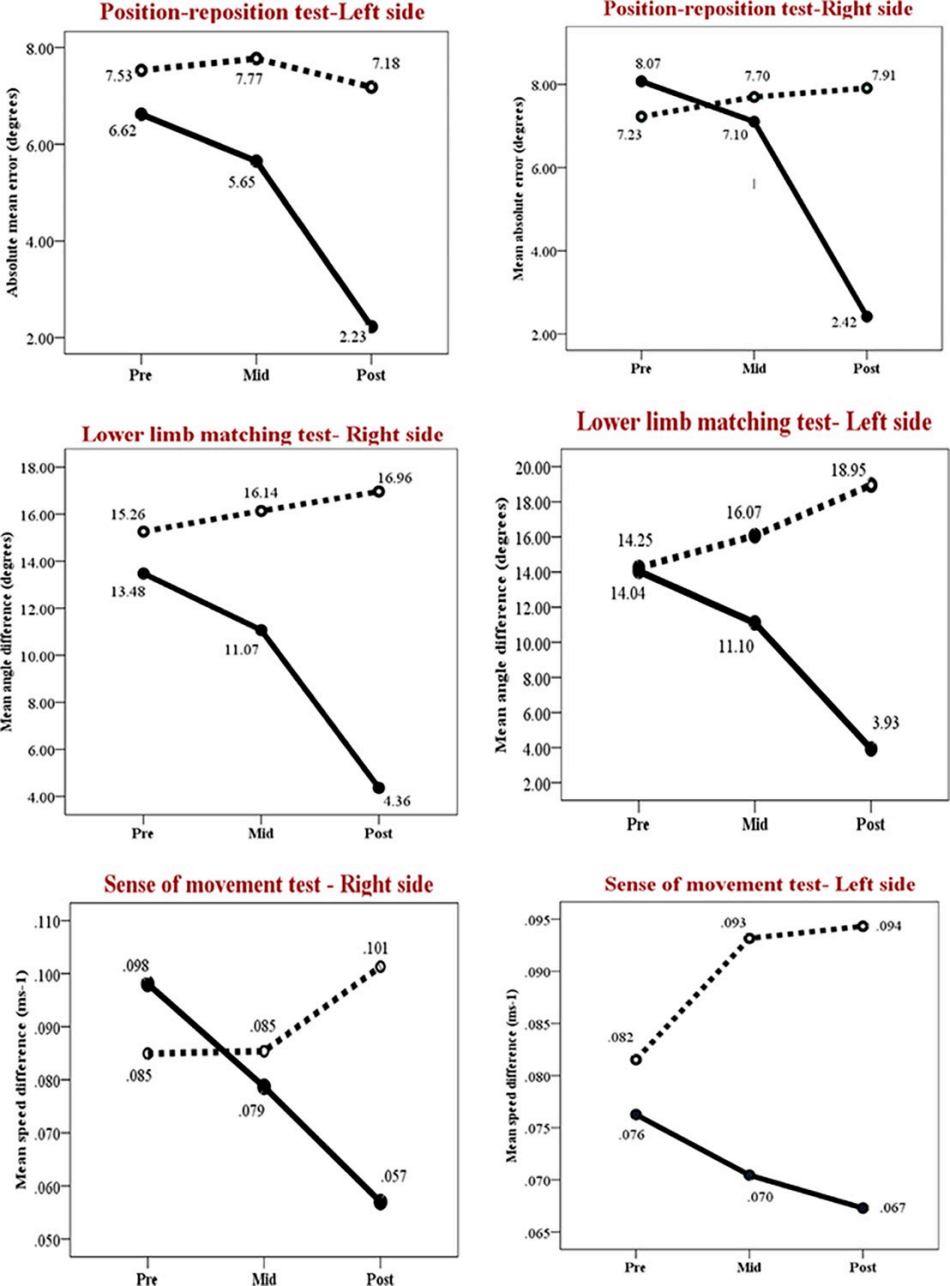
Pre-, mid and post-intervention mean differences for the lower limb (A) Position re-position test; (B) Lower limb matching test and (C) Sense of movement test in the intervention group and control group. Pre = Pre-intervention, Mid = Mid-intervention (at 6^th^ week) and Post = Post-intervention (at 12^th^ week).

The time*group interaction effects on lower limb static and dynamic proprioception of intervention and control group are presented in Fig 2.

### Effect of gender and side of the body on the outcome of PRP

Additionally, covariate analysis was conducted to test whether the effect of PRP on lower limb static and dynamic proprioception function statistically significantly different after adjusting the effect of two covariates i.e. gender and side. The results revealed that simple main effects of the covariates and interaction effects were not statistically significant (Table 3). The study found that the effect of the novel PRP does not vary depending on the gender and the side. Therefore, the final results were interpreted based on unadjusted, true mean differences of the outcome measures.

**Table 3:** Effects of intervention, time and interaction on static and dynamic proprioception.

Outcome measure	Two-way ANOVA effect		Covariate analysis		
	Time frame	Mean difference ± SE (*p* value)	Gender (Between-subject)	Side (Within- subject)	Side*Group (Within -subject)
			(p value)	(p value)	(p value)
*Position-reposition test*: *Absolute mean error (°)*					
Right	T1-T2	0.25±0.21 (0.75)	0.96	0.44	0.75
	T1-T3	2.48±0.39 (<0.001)*			
Left	T1-T2	0.36±0.22 (0.31)	0.44		
	T1-T3	2.37±0.48 (<0.001)*			
*Lower limb matching test*: *Mean angle difference (°)*					
Right	T1-T2	0.77 ±0.61 (0.66)	0.66	0.18	0.84
	T1-T3	3.71 ±0.82 (<0.001)*			
Left	T1-T2	0.56 ±0.56 (0.97)	0.14		
	T1-T3	2.23 ±1.07 (0.01)*			
*Sense of movement test*: *Mean speed difference (ms*^*-1*^*)*					
Right	T1-T2	35.78 ±12.09 (0.19)	0.40	0.12	0.16
	T1-T3	50.06 ±26.61 (0.21)			
Left	T1-T2	11.00 ±16.7 (1.00)	0.11		
	T3-T1	7.21 ±31.31 (1.00)			

*p < 0.05, compared with control group

*Note*. T1 = Baseline; T2 = 6 weeks following the intervention; T3 = 12 weeks following the intervention

## Discussion

This study examined (1) whether novel PRP improved lower limb static and dynamic proprioception compared to no exercise and (2) whether the effect of PRP varied across side i.e right vs left and gender i.e males vs females of patients with DPN. The study found a significant reduction of pre-post error for position-re position sense and lower limb matching test in both limbs and right side sense of movement test following 12-weeks of intervention which attributes to the improvement in static and dynamic proprioception sensation. Six weeks duration is not enough to obtain a significant improvement in proprioceptive deficits. None of the proprioceptive deficits improved in the control group. Effectiveness of the PRP dids not significantly differ based on the gender and side of the body.

### Pathophysiology of DPN

Microvascular dysfunctions in diabetes is indeed a widespread phenomenon which had been found to damage to nerves, vessels, retina, kidneys, skin, brain, heart, and lungs. It is hypothesized that elevated blood glucose level in DM induces microvascular endothelial dysfunction and impaired mitochondrial oxidative phosphorylation activity leading to diabetes-related microvascular complications. Furthermore, microcirculation associated with DM also contributes to the pathogenesis of microvascular complications of diabetes viz retinopathy, nephropathy, and neuropathy [20].

DPN is the presence of symptoms and/or signs of peripheral nerve dysfunction which cause damage to the sensory and motor nerves. Sensory and motor peripheral nerve dysfunction distinctly affects the physical performance in DPN patients. Loss of sensory nerve function leads to a reduction in sensory inputs from the extremities [46] whereas, loss of motor nerve function leads to insufficient innervation of lower limb muscles resulting in muscle weakness and atrophy [47]. Owing to the deficits of proprioception combined with motor impairments, which are key determinants for the motor control for functional independence, DPN patients present with diminished mobility and compromised quality of life leading to notable levels of morbidity [48, 49].

### Sensorimotor rehabilitation in DPN

Amongst the different interventions to treat DPN, exercises and rehabilitation are proven to be an promising tool to regain functional independence [50]. Interestingly, previous studies reported a reduction in premature activation of lower limb muscles in order to compensate for diminished sensory information [51, 52] and increased muscle activity [53] in response to exercises in patients with DPN.

Sensorimotor training is a comprehensive strategy which is found to be one of the effective non-pharmacological interventions to improve DPN-related outcomes [27]. The founder of sensorimotor rehabilitation, Janda et al. (1996) stressed increasing the proprioceptive input by improving afferent information into CNS to facilitate automatic coordinated movements [31]. Sensorimotor training involves progressive stimulation of sensory inputs through specifically designed exercises with increasing levels of challenge of the task using a functional, rather than a structural approach [31, 32]. Sensorimotor exercises that facilitate sensory inputs i.e proprioception are believed to facilitate motor program if sensorimotor exercises done at the beginning [33]. Also, a gradual increase in the difficulty of functional tasks leads to enhance proprioception function.

Previous studies have shown that sensorimotor training improves supraspinal reorganization [54], regeneration of neuromuscular structures [34] and reduce reflex excitability [55] leading to improved proprioception [54]. Further, Song et al. (2011) reported the improvement of trunk proprioception in patients with DPN following a specific balance training program [24]. However, it is doubtful whether these changes could be through the enhancement of proprioception function or not.

Nevertheless, there remains a lack of evidence supporting lower limb proprioceptive rehabilitation programs which specifically improves the key elements of proprioception viz static and dynamic proprioception in patients with DPN. The novel PRP could fill the gap by providing the scientific evidence to physiotherapists.

### Novel PRP

The rehabilitation protocol of the present study has been designed to intensify the proprioceptive sensory inputs through functional activities. The PRP adheres the basic principles for improving proprioception which is believed to be impaired due to the pathological process of DM. In fact, the rehabilitation program addresses the following key elements; participant’s concentration, rationalization, feedback, active exercises, and repetitions which act as adaptive codes to improve neuromuscular control by recruiting proprioceptors [45].

Participant’s concentration and rationalizing is the mental process in which the participant is aware of and attentive to the movement that he/she performs. Incorporation of cognition and rationalizing during each exercise is expected to improve the ability of decision-making and organizing responses not solely in typical tasks but also challenging activities. This adaptive code also assists in effective motor learning by recruiting proprioceptors thereby improving motor skills in every functional task. Kinaesthetic feedback is another method of engaging proprioceptors for motor learning; where the practitioner provides feedback on the accuracy, sequence and quality of the movement that the patient performs. This feedback is believed to optimize the learning of motor control. Active involvement in exercises is the other concept of neuromuscular adaptation as the motor system efficiently engages during active movements compared to passive or active assisted exercises. When the participant actively performs the exercise, they make mistakes which they learn from their own mistakes and try to correct them. This concept is also important in recruiting proprioceptors into motor learning and thereby improving postural control. Further, more repetitions of the same exercises are also thought to assist in the effective engagement of proprioceptors during motor learning [45].

The results of the present study provide evidence to state that the involvement of these concepts together with other concepts i.e increased demand, different practice settings, and longer dutaion i.e. 12 weeks makes the novel PRP more effective in regaining static and dynamic proprioceptive acuity in patients with DPN.

### Comparison of PRP with other sensorimotor rehabilitation programs

Few sensorimotor rehabilitation programs have been developed and their efficacy been studied on various functional outcomes in people with DPN. These sensorimotor rehabilitation programs include different types of exercises i.e wall slides, bridging, plank, sit-to-stand, wobble board exercises, one leg and double leg stance, heel and toe raise, tandem stance, balance training [36] which gradually progressed to different surfaces i.e firm to foam [36]. Gbiri et al. (2021) developed a safe, home-based protocol; *Lagos Neuropathy Protocol (LNP)* which was found to improve sensory perception, pain, strength, balance and functional performance in patients with DPN [56]. This protocol comprised 10 different exercises to work on different surfaces and textures i.e. foam, seeds, mat, cotton and wool.

These findings also agrees with the findings of Santos et al. (2003), who observed better improvement of tactile sensitivity and body-sway among women with DPN following 12 weeks of proprioceptive training [29]. During the program, patients performed exercises on various textures i.e foam, wood box with beans, cotton and sandpaper in different speeds. Additionally, balance board, proprioception ball and medicine balls were used to train the patients. The study found that the LL proprioception measured by means of plantar sensitivity was significantly improved by 46% and 85% following 6-week and 12-week of proprioceptive rehabilitation program respectively in women with DM [29].EI-Wishy et al. (2012) also involved in sensorimotor rehabilitation program consisted of 13 stations with different textures on which the patients performed the exercises, and it improved balance indices of the participants [28].

Furthermore, sensorimotor training combined with other exercise interventions were found to exert positive effects not only on lower limb sensation [36, 38] but also on improving various impairments associated with DPN, such as nerve dysfunction [36, 57], pain tolerance [57], muscular activation pattern [36], walking [36], and balance [38, 57]. Spatiotemporal parameters of gait such as velocity, stride length were found to be significantly improved following 8-week of sensorimotor rehabilitation program among middle-aged and older-aged patients with DPN [58].

As aforementioned sensorimotor rehabilitation were conducted combined with gait training, strength training and balance training,etc the sole effect of sensorimotor training on the improvement of the desired outcome may not directly be determined. The observed improvement could result either due to pure effects or combined effects of different exercise programs. The PRP developed in the current study confirms that the effect of PRP directly targets improving proprioceptive deficit in people with DPN. As the improved proprioception may result in correct motor programing, hence effective motor control of movement, further studies should investigate the effect of novel PRP on different aspects of physical activities i.e walking on different textures, slopes, obstacle navigation, etc. The reduction of functional capacity in DPN patients may largely due to the improper proprioception function. Hence the improvement of ADL following PRP is justified.

Similarities were also found in the previous studies. Most of the rehabilitation program were conducted for longer durations i.e over 8 weeks [36], 10 weeks [56] and 12 weeks [29] where the novel PRP was also carried out for 12 weeks. Even though, the 6-week duration of current PRP was found to be not adequate to elicit significant improvement of LL proprioception, a study conducted by Ahmad el al (2019) showed a significant improvement in trunk proprioception following 8-week of sensorimotor rehabilitation among middle and older patients with DPN [38].

While the aforementioned rehabilitation programs focused on improving functional outcomes, the current study investigated PRP as an effective intervention to improve lower limb static and dynamic proprioception. PRP involved simple functional exercises yet more advanced theoretical and evidence-based approach to directly address lower limb proprioceptive deficits hypothesizing that better proprioceptive acuity will serve better functional stability in each and every daily task. It is believed that improved lower limb proprioception provides a solid basis for every lower limb task making them more effective and efficient despite extravenous factors such as external perturbations, environmental alterations, etc. The novel PRP does not require any sophisticated equipment to perform the exercises yet it exerts significant improvement on lower limb proprioception errors. Therefore, it bestowed on clinicians that the rehabilitation should consist of exercises to address proprioceptive deficits caused by DPN thereby improving the functional level of patients with DPN. The novel PRP claim that cost-effective, safe, evidence-based protocol can be performed in the absence of any sophisticated equipment.

While previous studies have documented the effectiveness of various sensorimotor training protocols on balance, gait and functional performance, there is a dearth of literature on the effectiveness of those sensorimotor and/or proprioceptive training with respect to static and dynamic proprioceptive deficits in DPN. Further studies may be needed to explore the efficacy of PRP over conventional rehabilitation on functional independence in DPN patients.

Although a larger sample size may be needed to further confirm the efficacy of PRP in reducing disability in DPN patients, the outcome of this study depicted that PRP is more effective in improving static and dynamic proprioception compared to no exercises in DPN patients.

In fact, most patients do not have signs and symptoms at the earlier stages of DPN hence, the patient may be identified at the later stage where there are many other complications with the progression of DPN. Therefore, DPN patients should be routinely screened for proprioceptive deficits when they undergo foot examination in order to start the appropriate interventions and to avoid complications.

### Effect of sensorimotor rehabilitation for gender

Contradictory evidence was found in the literature regarding gender differences in proprioceptive deficits. One study suggests that females exhibit more proprioceptive deficits than males [59] whereas another study found that males have more proprioceptive deficits than females [60, 61]. This highlights the need to have the gender-related differences taken into account when developing rehabilitation programs specially aimed at proprioceptive deficits. However, the present study found that gender does not influence the efficacy of PRP, suggesting both males and females could get equal benefits from the developed PRP. Therefore, it is suggestive that the PRP equally benefits DPN patients irrespective of gender and the pre-test proprioception acuity level.

### Effect of sensorimotor rehabilitation for side of the body

Proprioception task performed by non-preferred/non-dominant side is found to be more accurate compared to the dominant side. It is been attributed to better utilization of proprioceptive information by the non-dominat hand compared to dominant hand based on role of each side in bimanual tasks [62]. Generally, the role of non-dominant side is to static stabilization of an object for the dominat arm to dynamically manipulate [63]. Therefore, non-dominant hand is specialized for static limb position control whereas dominant side is specialized for dynamic movement control [64]. When comparing the proprioception function between left and right sides, left side superiority was observed than right side in lower limb in healthy adults [65]. Concerning side-specific attributes of proprioception, a study found no side difference in static proprioception in knee joints [66]. Similarly, Symes et al. (2010) found a significant movement discrimination ability in the non-dominant left side [67]. Contrary evidence suggest that proprioception is quite site-specific, and it is not correlated with joints with a different pattern of uses [68].

Regarding DPN, it was found that DPN patients exhibit higher proprioceptive deficits compared to healthy adults [6, 7]. Studies conducted to compare proprioceptive dysfunction in patients with DPN also reported smaller total error scores in left side compared to right side [61] confirming the left side superiority.

However, the novel PRP improved lower limb static and dynamic proprioception irrespective of the side of the body i.e right vs left confirming that PRP has the potential to reduce the proprioceptive errors in both lower limbs despite its level of proprioception function. Since it is being observed that patients with DPN commonly experience proprioceptive deficits, healthcare practitioners might consider introducing 12-week PRP for patients exhibiting such deficits in their lower limbs. This recommendation stems from two main considerations: firstly, recognizing the potential impact of untreated proprioceptive deficits on daily functioning, and secondly acknowledging the notable positive impact that the novel 12-week PRP could have on addressing both static and dynamic proprioceptive deficits in the lower limbs.

Some limitations of this study have to be acknowledged. The study involved only a limited number of participants, as this is a pilot study of a large clinical trail. Hence, the power of the study is reduced. The sample comprised patients diagnosed with DPN, encompassing different severity levels and disability statuses. The improvement of lower limb static and dynamic proprioception was evaluated individually in comparison to a control group, without factoring in the DPN severity levels during the pre-test proprioceptive deficits assessment. Consequently, this study was unable to present the average improvement of proprioceptive deficits based on DPN severity levels. However, the findings of this study could be considered for further investigations of the effect of PRP in proprioceptive deficits in DPN patients considering their level of DPN severity and level of disability.

## Conclusion

This pilot study investigated the effectiveness of a novel, 12-week proprioceptive rehabilitation program on static and dynamic proprioception of both lower limbs in patients with DPN. However, this novel 12-week PRP yielded positive effects on proprioceptive deficits in both lower limbs in patients with DPN which significantly reduced errors for position re-position test and lower limb matching test solely in the intervention group. Future RCTs involving a large sample size, may compare the proprioceptive rehabilitation program with conventional physiotherapy to investigate the effectiveness on postural abnormalities, functional performance and quality of life in patients with DPN.

## Supporting information

S1 File Proprioceptive rehabilitation protocol. (PDF)

S2 File Trail study protocol. (PDF)

S3 File CONSORT checklist. (DOC)
