# Supplementary material for: A novel proprioceptive rehabilitation program: A pilot randomized controlled trail as an approach to address proprioceptive deficits in patients with diabetic polyneuropathy
Source: PLoS One. 2024 Jul 5;19(7):e0305055. doi: 10.1371/journal.pone.0305055 (PMC11226081; doi:10.1371/journal.pone.0305055)
Supplement: S1 File — (PDF) [file pone.0305055.s001.pdf]

Proprioceptive Rehabilitation Program

Station	Exercise intervention	Description	Time allocation
1.	**Warm up Session**<br>• Slow walking	Participant was instructed to walk around the room	**3 min**
	• Stroking and kneading	Participant was sitting with legs crossed and performed slow strokes over the anterior and posterior aspect of leg, in the distal to proximal direction using pads of the fingers (from the tip of the toes to knee) followed by circular kneading over the toes and foot using the pads of the thumbs (from the tip of the toes to ankle).	**3 min**
	• Cyclical joint movement and oscillations	Participant was instructed to slowly move his/her ankle in an upward-downward direction, clockwise and anticlockwise while sitting on a chair.	**2 min**<br>3 repetitions* 3 sets in each direction<br>5 seconds rest between each set
	• Stretching of calf muscle groups	Participant was instructed to stand while bending the front leg and keeping the back leg straight. Then the patient was instructed to lean forward until he/she felt a stretch on calf muscles and keep that position for 30 sec and release.	**4 min**<br>3 repetitions* 1 set for each muscle group<br>30 seconds rest between each repetition and each set
2.	**Spatial orientation**	Participant was instructed to move his/her lower limb between two targets with eyes opened.	**4 min**

		Progression: Perform the same task with eyes-closed, increased distance and speed.	
3	**Static Balance**	Participant was instructed to stand and perform different simple daily tasks using upper limb.<br><br>*Progression:* Perform various tasks with varying difficulties on various platforms i.e floor, foam cushion, increased base of support, and eyes-closed.	**4 min**
4	**Dynamic Balance – Foam cushion exercise**	Participant was instructed to stand on foam cushions in different stances.<br><br>Progression: Perform the exercises with eyes-closed, reduced the base of support and assistance.	**4 min**
5	**Stability Challenge**	Participant was standing and instructed to move one hand between two positions in different directions marked by the Physiotherapist's both hands.<br><br>Progression: Performed the same exercise on different platforms with eyes-closed, changed the direction, distance and speed of arm movements.	**4 min**
6	**Reaction time**	Participant was sitting on a high couch and instructed to keep his/her ankle plantar flexed at 30^0. Physiotherapist applied a force against the position and suddenly removed the hand and the participant was instructed to maintain the ankle in position at the same angle. The same procedure was performed with ankle dorsiflexed at 10^0.	**2 min**<br><br>3 repetitions* 2 sets for each joint<br><br>30 seconds rest between each set

		Progression: Perform the same exercise with eyes-closed.	
7	**Fine Motor Skills**	Participant was instructed to perform simple different tasks using toes and foot.<br><br>Progression: Moved the leg in space in the direction of different numbers, shapes, letters of English alphabet, letters of Sinhala alphabet and clock times. Picked up a few objects (pen, small stone, piece of cloth, chalk, bottle lid) placed on the floor from the toes and put them into the box, one object at one time.Performed the exercise with eyes-closed.	**3 min**
8	**Time up and go**	Participant was sitting on a chair and instructed to stand up upon physiotherapist's command and walk 3 meters ahead, turn around and walk back to the chair.<br><br>Progression: Navigate obstacles in the walkway, walk on the foam cushion placed in the walkway,	**3 min**<br><br>3 repetitions* 1 sets<br><br>30 seconds rest between each repetition
9	**Cool down exercises**<br>• Slow walking	Participant was instructed to walk around the room	**2 min**
	• Stretching of calf muscle groups	Participant was instructed to stand while bending the front leg and keeping the back leg straight. Then the patient should lean forward until he/she feels a stretch in calf muscles. Participant should keep that position for 30 sec and release.	**4 min**
		**Total time duration**	**40-45 minutes**
