# Supplementary material for: A novel proprioceptive rehabilitation program: A pilot randomized controlled trail as an approach to address proprioceptive deficits in patients with diabetic polyneuropathy
Source: PLoS One. 2024 Jul 5;19(7):e0305055. doi: 10.1371/journal.pone.0305055 (PMC11226081; doi:10.1371/journal.pone.0305055)
Supplement: S2 File — (PDF) [file pone.0305055.s002.pdf]

# Study Protocol

## Research Design

This interventional study was designed as a double-blinded, randomized controlled to assess the effectiveness of the proprioceptive rehabilitation program (PRP) on lower limb static and dynamic proprioception in the patients with diabetic polyneuropathy (DPN). This study was conducted as Stage I of a larger RCT to investigate the efficacy of proprioceptive rehabilitation in improving lower limb proprioceptive deficits in DPN patients which was then compared with the conventional rehabilitation. There were no adverse effects that arose from the proprioceptive rehabilitation program.

## Study Site

The target population of this study was the patients who had type-2 DM along with DPN. Patients who regularly visited the Diabetic and Endocrinology clinic at National Hospital, Kandy were recruited to the study under the supervision of Dr. Charles Antonypillai, Consultant Endocrinologist, Diabetic and Endocrinology Clinic, Kandy National Hospital, Sri Lanka.

## Inclusion Criteria and Exclusion Criteria

### *Inclusion Criteria*

- Having type-2 DM for one or more than one year
- Diagnosed with DPN
- Within the age group of 35-60 years
- Provided informed written consent to participate in the study

### *Exclusion Criteria*

- Patients with a history of cardiovascular diseases, recent surgery, severe pain and paresthesia, other types of neuropathy, diabetic foot ulcers, impaired vision,

autonomic dysfunctions, significant psychiatric disorders, amputation, pregnant women, and obese patients

- Patients and who were not willing to give consent were excluded

**Sampling Method**

An unbiased sample of thirty patients was obtained from the patients who regularly visited Diabetic and Endocrinology Clinic. The selected patients randomly allocated either to the *intervention group* (n=15) and *control group* (n=15) using concealed envelop randomization. For this randomization, the random allocation sequence was generated and it remained concealed in sequentially numbered, opaque envelopes which were assignment sealed. Patient drew a number in sequence and patient's reference number was noted on the envelope before tearing it. Lastly, the envelop was opened to disclose the treatment allocation. The patient and the assessor were blind from group allocation.

**Variables**

- Diabetic polyneuropathy
- Lower Limb static and dynamic proprioception

**Data Collection and Data Collection Tools**

Ethical clearance for this study was obtained from Ethics Review Committee, Faculty of Medicine, University of Peradeniya (2023/EC/20), and it was registered under Sri Lanka Clinical Trial Registry with the registration number of SLCTR/2023/017 to conduct as a clinical trial. Patients were recruited from the Diabetic and Endocrinology Clinic at National Hospital Kandy in October 2023. One hundred and twenty patients with type-2 DM were screened using validated and reliability tested Sinhala and Tamil versions of Michigan

Neuropathy Screening Instrument (MNSI).[1,2] During the screening, the self-administered questionnaire (Part A) of MNSI was distributed among the patients who were at the clinic. Patients with DPN symptoms who scored greater than 4 in the self-administered questionnaire of MNSI were selected.[3,4,5] Then, the physical examination part (Part B) of the MNSI was conducted among the selected patients to confirm the presence of DPN. Patients who scored greater than 2 points in the physical assessment part of MNSI were diagnosed with DPN and hence recruited to the study.[1,3,6] This method was applied to the pre-defined group until the required study sample of 30 patients was fulfilled.

After obtaining informed written consent from the eligible participants, demographic data i.e., age, sex, type and duration of diabetes were recorded. Then, the participants were randomly allocated into either the *intervention group (n=15)* or the *control group (n=15).*

This study was designed to assess the efficacy of a specially designed, evidence-based, novel proprioceptive rehabilitation program (PRP) in improving static and dynamic proprioception in lower limbs compared to no intervention in patients with DPN. Intervention group underwent PRP for 45 minutes of duration, thrice a week for 12 weeks. The control group received no exercises. Both groups had regular diabetic care and attended the clinic continuously. Participants who were unable to follow the program continuously for more than three consecutive weeks were disqualified from being a subject in the study. Proprioception of both the lower limbs was assessed by static and dynamic proprioception functions [1,7] at the baseline, at the 6th week (mid-assessment) and at the 12th week (post-test assessment) by an independent assessor who was blind to the group allocation.

**Outcomes of interest and outcome measuring tools:**

- Position-reposition test to assess the static position sense at ankle joint

- Lower limb matching test to assess the ability of the patient to perceive the knee joint movement
- Sense of movement test' to assess the ability of the patient to perceive the lower limb movement

**Tests and Measurements:**

Static position sense and dynamic movement sense were assessed in both the legs to identify the lower limb proprioception function in each participant at the baseline, 6 weeks after the commencement of rehabilitation program and at the completion of 12-week rehabilitation program in intervention group and without any intervention in control group.

Prior to each test, participant was familiarized with the particular tests by giving clear and accurate instructions and demonstrations. Visual and auditory cues were eliminated during proprioception assessments.

*Measurement of Static Position Sense*

'Position-reposition test' was used to evaluate the static position sense in both ankle joints which assessed the ability of the patient to match input from muscle and joint proprioceptors.

'Position-reposition test' was conducted as follows (Figure 1A):

i. Participant seated in a high-sitting position on a wooden stool with eyes closed, the knees flexed at 90° and the lower leg positioned vertically.

ii. Participant's ankle joint was set to a pre-determined position of 30° of plantar flexion (target position) by the investigator.

iii. The position was held for 2-4 seconds by the investigator which allowed the participant to feel and remember the target position followed by passively moving the ankle into

starting position. Participant was then instructed to reproduce the angle at the same ankle joint from the neutral position.

iv. The difference between the target position and the reproduced position[8] was measured using goniometer in Kinovia movement analysis software to identify the accuracy of the static position sense (Figure 1B)

v. Same procedure was repeated with second predetermined position of 10° of dorsiflexion to the same leg.

vi. Participant performed three trials in each leg and each trial was followed by 15-second rest period.

vii. Absolute mean error (i.e. the absolute difference between the target and reproduced angles) of three trials were recorded to the nearest 0.01 ° degrees as the measure of static position sense in right and left ankle joints. Prior to each test, participant performed three practice trials to get familiarized with the test.

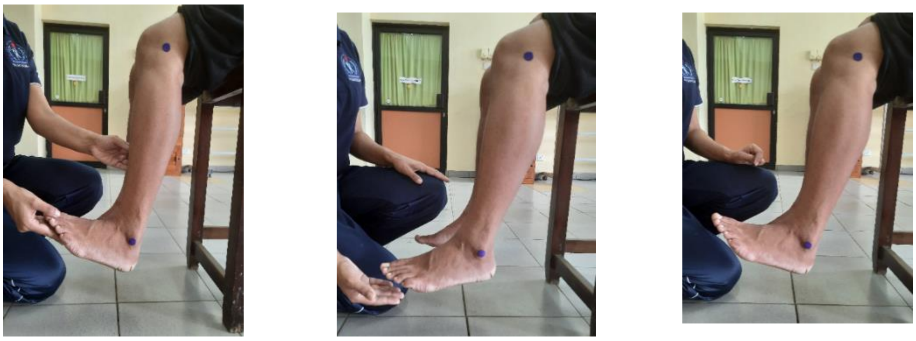

**(A)**

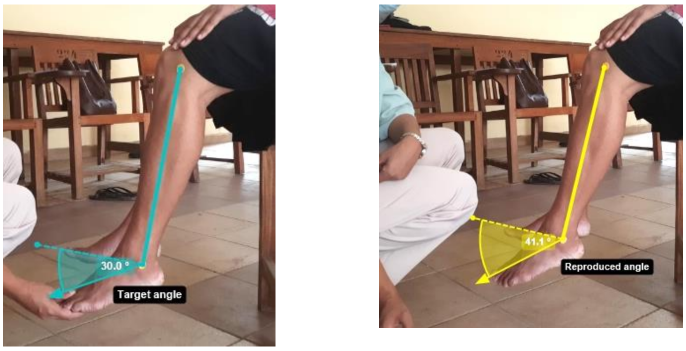


**(B)**

**Figure 1** (A) Performing 'Position re-position' test to assess lower limb static proprioception. (B) Obtaining target angle and reproduced angle for 'Position re-position' by the goniometer using kinematic analysis software

*Measurement of Dynamic Movement Sense*

'Lower limb matching test' and 'Sense of movement test' (Lederman, 1991) were carried out to determine the ability of the patient to perceive the lower limb movement sense.

The tests were conducted as follows :

i. Participant seated in a high-sitting position on a wooden stool with eyes closed, the knees flexed at 90° and the lower leg positioned vertically.

ii. Investigator moved the participant's lower limb forward and backward (flexion and extension) in different speed and instructed the participant to follow the same movement with the contralateral leg at the same speed and same direction.

iii. The difference between target (ipsilateral) and reproduced (contralateral) knee joint angles were recorded to the nearest 0.01° degrees using Goniometer in Kinovia movement analysis software (Figure 2).

iv. The mean difference between the target (ipsilateral) and reproduced (contralateral) knee angles in three trails were calculated to obtain the 'mean angle difference' as the outcome measure of the 'Lower Limb Matching Test'

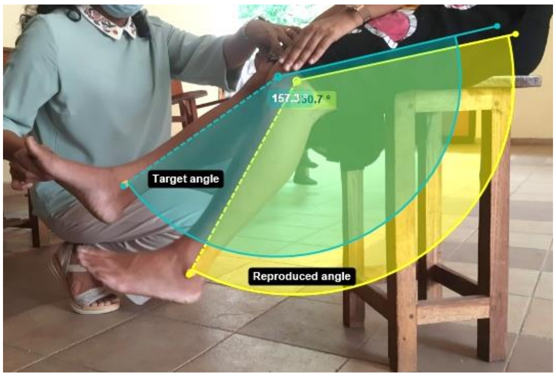


**Figure 2.** Measuring target angle and reproduced angle for 'Lower Limb Matching Test' using kinematic analysis software

v. Similarly, the mean difference of the speed between ipsilateral leg and contralateral leg in three different random positions was calculated to obtain 'mean speed difference' as the outcome measure of the 'Sense of Movement Test' (Figure 3).

vi. The mean speed difference of three trails was obtained from pixels per second (px/s) which was then transformed to meters per second (m/s) to report the ability to detect movement of lower limb by lower limb proprioceptors. (1px= 0.0002645833m). (in results)

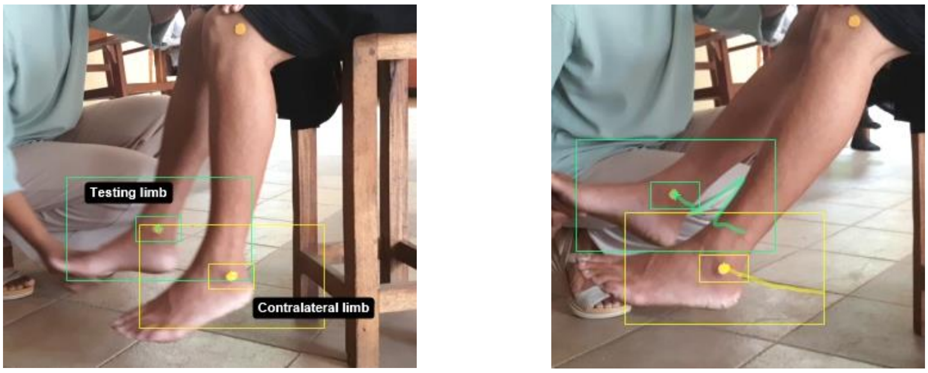


**(A)**

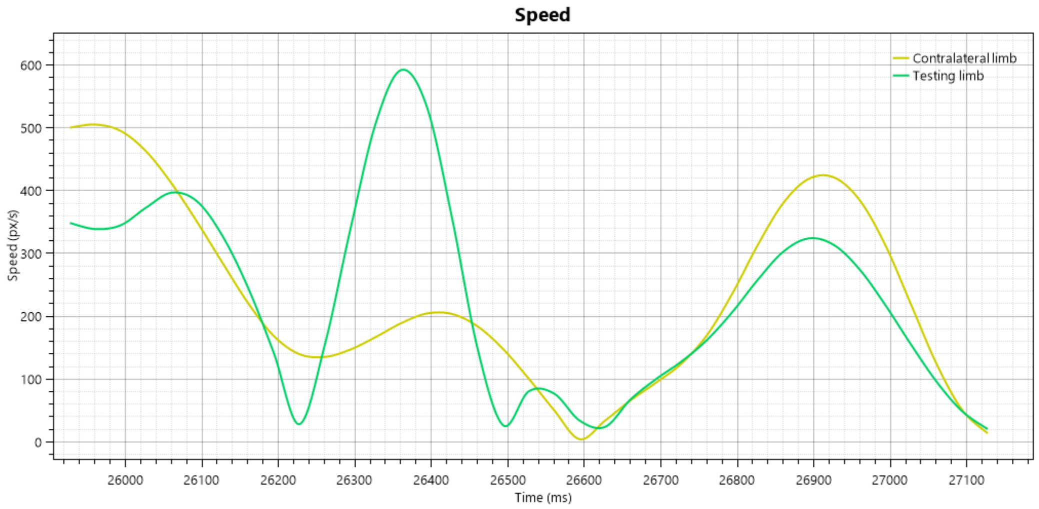


**(B)**

**Figure 3.** (A) Obtaining speed difference for ‘Sense of movement test’ (B) Output graph indicating lower limb speed of testing limb and contralateral limb

Static and dynamic proprioception tests procedures were recorded using a high-speed video camera in the sagittal plane with the test limb facing the camera. The videography was taken at the rate of 30 frames per second with a screen resolution of 720 × 1080 pixels in an MP4 file. The camera was mounted on a tripod which was set on automatic focus. To facilitate the identification of anatomical landmarks on the video, the human body markers with a 1.5 cm diameter were placed on the greater trochanter, lateral epicondyle, and lateral malleolus of the participants' test limbs.

Kinovea 0.9.5 motion analysis software (Joan Charmant & Contributors, 2006) was then used to analyze and record the target angle and reproduced angles formed by lines connecting the centers of the body markers in degrees to the nearest 0.1◦. Similarly, the speed of each lower limb during the test was evaluated using the linear kinematic analysis option in Kinovea motion analysis software.

The mean of three trials of each dynamic movement sense was considered to be representative of the global proprioception function in which the higher error values corresponded to the lower proprioception function.

**Proprioceptive Rehabilitation Program**

A properly designed proprioceptive rehabilitation program was conducted among the participants of the intervention group. This novel proprioceptive rehabilitation program was developed by the researcher based on the latest available evidence under the guidance of the experts in the field of physiotherapy**.** Each session of this specialized rehabilitation program was comprised of different types of exercises to restore the proprioceptive deficits. The proprioceptive rehabilitation protocol is attached in Annexure 01.

Each session of the proprioceptive rehabilitation program included three sub-sessions: (1) Warm-up session, (2) Proprioceptive rehabilitation session and (3) Cool down session. The whole rehabilitation program adhered to the basic principles for improving proprioception and it addressed the key elements of proprioception which was suspected to be affected by the disease process of DM. Accordingly, the exercises of the proprioceptive rehabilitation program were designed to address the following key elements; participant's concentration, rationalization, feedback, active exercises, and repetitions to improve postural stability.[9,10, 11,12] All these elements act as adaptive codes to improve neuromuscular adaptation by recruiting proprioceptors to improve postural instability.

The progression of each exercise was carried out by modifying the movement parameters i.e. force, length, velocity and endurance of each exercise. The exercise was further challenged by reducing base of support (BOS), increasing center of gravity (COG) sway on BOS, changing the surface on which the participant performs the exercises, engaging upper limb tasks while

maintaining balance, introducing different obstacles in the walkway and removing visual cues by instructing the participant to perform some exercises with eyes closed.

Closed kinetic chain exercises which were performed on the firm surface i.e. floor and foam surface i.e. cushion was designed to improve neuromuscular control of lower limb muscles through proprioceptors. Single leg standing exercises was designed to challenge the lower limb muscles and recruit the neuromuscular system for proximal stabilization at the hip, which was essential in maintaining postural stability during different tasks. Exercises designed to perform simultaneous activities of the upper limb while the participant balances on lower limbs helped to challenge the neuromuscular pathways by effectively engaging proprioceptors for motor control. Despite the included exercises resembled ordinary exercises, each component of the exercise designed and modified based on the basic concepts of improving impaired proprioception.

A proper warm-up and cool down sessions were conducted before and after each rehabilitation session to improve the effectiveness of the exercise program as well as to reduce the incidence of injuries. One session of the proprioceptive rehabilitation program was carried out for 40-45 minutes, 3 days per week for 12 weeks.

The rehabilitation protocol was devised to increase the difficulty of the exercises every 2 weeks throughout the designated 12-week period. As the training progressed, each exercise was modified to make them more demanding by removing visual feedback (eyes closed) and alteration the support surface, body position, direction, speed, and distance.

**Statistical Analysis**

Data from 30 participants from intervention group (n=15; 6 males, 9 females) and control group (n=15; 4 males, 11 females) were included in the analysis. Absolute mean difference for position repositioning test, mean angle difference for lower limb matching test and mean speed

difference for sense of movement tests were obtained at three-time points i.e., baseline, 6 weeks and 12 weeks were compared between the intervention and control groups to investigate the effectiveness of the novel proprioceptive rehabilitation program on proprioception compared to no intervention.

Baseline data were compared between two groups to investigate the normality and homogeneity of variance across all the variables using Shapiro-Wilk test and Levene's test respectively. Two-way, group (intervention vs. control) × time (pre vs. post) repeated measure analysis of variance (ANOVA) was conducted on outcome measures related to static and dynamic proprioception to validate the novel proprioceptive rehabilitation program as a therapeutic tool to improve lower limb proprioception. Further, post-hoc analysis were performed to investigate whether the treatment duration i.e., 6 weeks and 12 weeks influence the improvement of lower limb proprioception. The level of significance was ascertained at a cut off $p$ value of less than 0.05. All statistical analyses were performed using IBM SPSS 22.0 software (Armonk, NY: IBM Corp).

**Annexure 1- Proprioceptive Rehabilitation Program**

| Station | Exercise intervention | Description | Time allocation |
|---|---|---|---|
| 1. | **Warm up Session**<br>• Slow walking | Participant was instructed to walk around the room | **3 min** |
| | • Stroking and kneading | Participant was sitting with legs crossed and performed slow strokes over the anterior and posterior aspect of leg, in the distal to proximal direction using pads of the fingers (from the tip of the toes to knee) followed by circular kneading over the toes and foot using the pads of the thumbs (from the tip of the toes to ankle). | **3 min** |
| | • Cyclical joint movement and oscillations | Participant was instructed to slowly move his/her ankle in an upward-downward direction, clockwise and anticlockwise while sitting on a chair. | **2 min**<br>3 repetitions* 3 sets in each direction<br>5 seconds rest between each set |

| | | | |
|---|---|---|---|
| | • Stretching of calf muscle groups | Participant was instructed to stand while bending the front leg and keeping the back leg straight. Then the patient was instructed to lean forward until he/she felt a stretch on calf muscles and keep that position for 30 sec and release. | **4 min**<br><br>3 repetitions* 1 set for each muscle group<br><br>30 seconds rest between each repetition and each set |
| 2**.** | **Spatial orientation** | Participant was instructed to move his/her lower limb between two targets with eyes opened.<br><br>*Progression:* Perform the same task with eyes-closed, increased distance and speed. | **4 min** |
| 3 | **Static Balance** | Participant was instructed to stand and perform different simple daily tasks using upper limb.<br><br>*Progression:* Perform various tasks with varying difficulties on various platforms i.e floor, foam cushion, | **4 min** |

| | | | |
|---|---|---|---|
| | | increased base of support, and eyes-closed. | |
| 4 | **Dynamic Balance – Foam cushion exercise** | Participant was instructed to stand on foam cushions in different stances.<br><br>Progression: Perform the exercises with eyes-closed, reduced the base of support and assistance. | **4 min** |
| 5 | **Stability Challenge** | Participant was standing and instructed to move one hand between two positions in different directions marked by the Physiotherapist's both hands.<br><br>Progression: Performed the same exercise on different platforms with eyes-closed, changed the direction, distance and speed of arm movements. | **4 min** |
| 6 | **Reaction time** | Participant was sitting on a high couch and instructed to keep his/her ankle plantar flexed at $30^0$. Physiotherapist applied a force against the position and suddenly removed the hand and the participant was instructed to maintain the ankle in position at the same angle. | **2 min**<br><br>3 repetitions* 2 sets for each joint<br><br>30 seconds rest between each set |

| | | | |
|---|---|---|---|
| | | The same procedure was performed with ankle dorsiflexed at $10^0$.<br><br>Progression: Perform the same exercise with eyes-closed. | |
| 7 | **Fine Motor Skills** | Participant was instructed to perform simple different tasks using toes and foot.<br><br>Progression: Moved the leg in space in the direction of different numbers, shapes, letters of English alphabet, letters of Sinhala alphabet and clock times. Picked up a few objects (pen, small stone, piece of cloth, chalk, bottle lid) placed on the floor from the toes and put them into the box, one object at one time.Performed the exercise with eyes-closed. | **3 min** |
| 8 | **Time up and go** | Participant was sitting on a chair and instructed to stand up upon physiotherapist's command and walk 3 meters ahead, turn around and walk back to the chair. | **3 min**<br><br>3 repetitions* 1 sets<br><br>30 seconds rest between each repetition |

| | | | |
|---|---|---|---|
| | | Progression: Navigate obstacles in the walkway, walk on the foam cushion placed in the walkway, | |
| 9 | **Cool down exercises**<br>• Slow walking | Participant was instructed to walk around the room | **2 min** |
| | • Stretching of calf muscle groups | Participant was instructed to stand while bending the front leg and keeping the back leg straight. Then the patient should lean forward until he/she feels a stretch in calf muscles. Participant should keep that position for 30 sec and release. | **4 min** |
| | | **Total time duration** | **40-45 minutes** |

## References


1. Feldman, E. L., Stevens, M. J., Thomas, P. K., Brown, M. B., Canal, N., & Greene, D. A. (1994). A practical two-step quantitative clinical and electrophysiological assessment for the diagnosis and staging of diabetic neuropathy. *Diabetes Care*, *17*(11), 1281-1289. https://doi.org/10.2337/diacare.17.11.1281
2. Nisansala, M. W. N., & Wimalasekera, S.W. (2015). Usefulness of Michigan Neuropathy Screening Instrument (MNSI) in Diagnosis of Diabetic Neuropathy in a Sri Lankan Diabetic Clinic. Proceedings of the Scientific Sessions of the Faculty of Medical Sciences, University of Sri Jayewardenepura, Sri Lanka
3. Abuzinadah, A. R., Alkully, H. S., Alanazy, M. H., Alrawaili, M. S., Milyani, H. A., AlAmri, B., . . . Bamaga, A. K. (2021). Translation, validation, and diagnostic accuracy of the Arabic version of the Michigan neuropathy screening instrument. *Medicine (Baltimore)*, *100*(44), e27627. https://doi.org/10.1097/md.0000000000027627
4. Aktar Reyhanioğlu, D., Adiyaman, S. C., Bektaş, M., Bulut, O., Özgen Saydam, B., Bayraktar, F., & Kara, B. (2020). Validity and reliability of the Turkish version of the Michigan Neuropathy Screening Instrument. Turkish journal of medical sciences, 50(4), 789–797.
5. Herman, W. H., Pop-Busui, R., Braffett, B. H., Martin, C. L., Cleary, P. A., Albers, J. W., & Feldman, E. L. (2012). Use of the Michigan Neuropathy Screening Instrument as a measure of distal symmetrical peripheral neuropathy in Type 1 diabetes: results from the Diabetes Control and Complications Trial/Epidemiology of Diabetes Interventions and Complications. *Diabet Med*, *29*(7), 937-944. https://doi.org/10.1111/j.1464-5491.2012.03644.x
6. Barbosa, M., Saavedra, A., Severo, M., Maier, C., & Carvalho, D. (2017). Validation and Reliability of the Portuguese Version of the Michigan Neuropathy Screening

Instrument. *Pain Practice*, *17*(4), 514-521. https://doi.org/https://doi.org/10.1111/papr.12479

7. O'Sullivan, S.B., Schmitz, T.J. and Fulk, G.D. (2014.) *Physical Rehabilitation*. 6th edition, Philadelphia. F.A.Davis Company. p:1020-1022.
8. Dover, G.& Powers, M.E. (2003). Reliability of joint position sense and force-reproduction measures during internal and external rotation of the shoulder. *Journal of Athletic Training*, 38 (4): 304-310.
9. Lederman, E. (2010). *Neuromuscular rehabilitation in manual and physical therapies* (4th ed.). Elsevier.
10. Holding, D.H. (1965). *Principles of training.* London: Pergamon Press.
11. Lee, T. D., Swanson, L. R., & Hall, A. L. (1991). What is repeated in a repetition? Effects of practice conditions on motor skill acquisition. *Phys Ther*, *71*(2), 150-156. https://doi.org/10.1093/ptj/71.2.150
12. Aman, J. E., Elangovan, N., Yeh, I. L., & Konczak, J. (2014). The effectiveness of proprioceptive training for improving motor function: a systematic review. *Front Hum Neurosci*, *8*, 1075. https://doi.org/10.3389/fnhum.2014.01075
